# Why Do Leaders Express Humility and How Does This Matter: A Rational Choice Perspective

**DOI:** 10.3389/fpsyg.2019.01925

**Published:** 2019-08-21

**Authors:** JianChun Yang, Wei Zhang, Xiao Chen

**Affiliations:** ^1^School of Business Administration, Guizhou University of Finance and Economics, Guiyang, China; ^2^The Pearl River Hydraulic Research Institute, Guangzhou, China; ^3^School of Business, Guangdong University of Foreign Studies, Guangzhou, China

**Keywords:** leader expressed humility, follower capability, inferred motives of leader expressed humility, trust, rational choice theory

## Abstract

The utility of leader humility expressing behavior has been examined by several studies across multiple levels. However, our knowledge about why leaders express humility continues to be sparse. Drawing on rational choice theory, this paper proposes a model examining whether followers’ capability triggers leader’s humility expressing behavior and how followers’ interpretations of it influence its effectiveness. Results from 278 leader-follower dyads from a time-lagged research design showed that followers’ capability as perceived by the leader is positively related to leader-expressed humility and, in turn, this behavior would conditionally enhance follower trust, that is, followers will trust the humble leader less when they attribute leader’s expressed humility more to serving impression management motives. Several theoretical and practical implications of this observation are discussed in this study.

## Introduction

Since Owens and Hekman (2012) developed a theoretical model of humble leader behavior, leaders’ humility expressing behavior has been receiving growing attention from leadership scholars (Owens and Hekman, 2012). Several investigators have confirmed the effectiveness of leader expressed humility across multiple levels—offering strategic value to firms (Vera and Rodriguez-Lopez, 2004), fostering better team climates (Owens and Hekman, 2016), legitimizing follower growth and development (Owens and Hekman, 2012) and reinforcing employee learning orientation, job satisfaction, work engagement, and performance (Owens et al., 2013, 2015).

Notwithstanding the positive outcomes alluded to the above, we still have only a vague understanding of why leaders enact humble behavior in organizations. Following classic ways of analyzing the causality of leader behavior, the antecedents of leader behaviors can be studied in terms of two sets of factors: personal and situational (Tuncdogan et al., 2017). In the cases of humble leader behavior, scholars have invested most of their attention into what personal factors impact a leader’s humble behavior, such as leader expressed humility. For example, previous studies showed that personal traits such as narcissism and honesty-humility were related to expressed humility (Owens et al., 2013); other individual differences such as learning goal orientation, relational identity and leader incremental theory of the self are also predictors of leaders’ humble behaviors (Owens et al., 2013; Wang L. et al., 2018). Another perspective emphasizes the importance of situational factors in affecting various leader behaviors, e.g., environmental strength (e.g., crisis) and organizational structure (Tuncdogan et al., 2017). Unfortunately, empirical research scrutinizing the situational antecedents of leader expressed humility is scarce. Only one study showed that team voice might predict leader expressed humility (Wang D. et al., 2018). This study indicates that characteristics of followers can be potential enablers of humble leader behavior.

Since there has been little effort put into exploring the situational predictors of leader expressed humility, our knowledge about leader humility continues to be limited, especially regarding how to cultivate humility in organizations. To reach a better understanding of leader humility, the first purpose of the present study is to explore the situational predictors of leader humility. Drawing from rational choice theory (Coleman and Fararo, 1992), we propose that leaders’ decisions in favor of expressing humility are the results of a rational calculation that they can indeed benefit from such specific actions. Specifically, since capable followers offer more benefits for the leaders and the team, we propose that the capability of followers (a type of situational factor) can act as a predictor of leader expressed humility (Tepper et al., 2011; Walter et al., 2015).

In addition, leaders’ rational consideration of whether to express humility raises further interesting questions such as what would happen if followers generate different interpretations of leader expressed humility and how they would then react. Giving consideration to this problem is important because employees can have varying interpretations of a leader’s behavior (Martinko et al., 2007; Sue-Chan et al., 2011), and thus they ascribe different motives behind expressions of humility exhibited by leaders (Owens et al., 2013). Dasborough and Ashkanasy (2002) proposed that followers’ interpretations of the leader’s behaviors would ultimately influence leadership outcomes. Therefore, our second purpose is to investigate how employees react to leader-expressed humility when they become aware of different motives of leader humility (i.e., performance enhancement motives or impression management motives). Following rational choice theory (Coleman and Fararo, 1992), we believe that followers’ trust building toward the leader will be influenced by followers’ interpretation of leader expressed humility (Zaheer et al., 1998). Specifically, when a leader’s humble behaviors are interpreted as being driven by impression management rather than performance enhancement, his/her followers would be less likely to engage in trusting relationships with the leader because when subordinates attribute leaders’ behaviors to impression management, they might be suspicious of the real motives behind leader behaviors (Bass and Steidlmeier, 1999; Li et al., 2017).

We hope to make contributions to the literature on leader humility and leadership along the following lines. Firstly, our study is the first to empirically investigate the influence of situational predictors on leader-expressed humility at the dyadic level, which would help foster a better understanding of why leaders express humility. Although a previous study showed that situational factors such as team voice might trigger humble leader behavior (Wang D. et al., 2018), this study neglected that humble leader behavior may occur within dyadic interpersonal interaction. Considering Owens et al. (2013) proposed that humble leader behavior connotes the interpersonal interactions between leaders and followers, we think the interaction targets’ characteristic (followers’ capability) may also impact humble leader behavior. By examining the predictive effect of followers’ capability on leaders’ humble behavior, our results thus provide further evidence that followers can serve not only as reactors but can also play a role in constructing leader behavior (refer to follower-centric leadership, Uhl-Bien et al., 2014).

Second, drawing on rational choice theory underpinning trust building, our research is one of the first to investigate followers’ interpretation of leader humility. Rational choice theory posits that the rational attribution about another’s behaviors is critical for trust building during social interactions (Weber et al., 2004; Whipple et al., 2013). Extending this opinion to the current study, we can infer that members’ attribution about leader-expressed humility might affect followers’ trust in leaders. Indeed, Martinko and Gardner (1987) pointed out that members’ attributions about leader behaviors play important roles in shaping leader-follower interactions. For example, previous studies showed that when members attribute leader transformational behaviors or ethical behaviors as serving impression management motives, followers will perceive their leaders in a less positive way (Dasborough and Ashkanasy, 2002; Li et al., 2017). Based on this line of research, our study hopes to contribute novel understandings on the effects of humble leader behavior through incorporating followers’ attribution of leader behavior to the humble leadership process. This perspective also provides empirical evidence for Owens and Hekman’s (2012) theoretical model which suggested that followers’ interpretations about leaders’ humble behavior works as the potential boundary condition for effective humble behavior from leaders.

**FIGURE 1:**
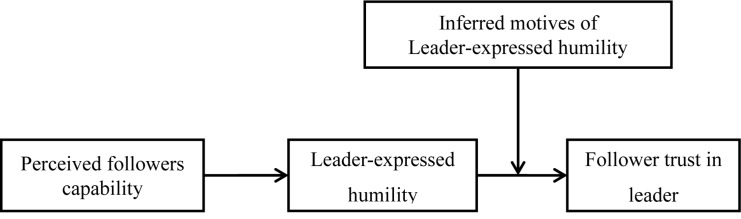
Integrative conceptual model.

Finally, we contribute to the literature of trust in leadership by integrating rational choice theory with the trust development process during leader-follower social interactions. Owens et al. (2013) suggested that expressed humility is a type of interpersonal interaction that involves at least two persons, such as one leader-follower dyad; such social interaction will help us to achieve a better understanding of why followers trust in leaders. Most previous studies only inspect the trust development from single perspectives, on one hand, leaders’ ability or benevolent behaviors (e.g., transformational behavior and supporting behavior) will impact follower trust in leaders (Burke et al., 2007); on the other hand, followers’ attributes, such as their propensity to trust and attribution style also impact trust in leaders (Nienaber et al., 2015). Given that trust is essentially generated from social interaction between the trustor and the trustee (Weber et al., 2004), we will integrate rational choice theory with the dyadic interaction as a way to understand why followers develop trust in leaders. This we do by clarifying the process through which followers’ capabilities influence leader expressed humility, which represents the leaders’ rational choice in the dyadic leader-follower interaction. Leaders’ humility expression, in turn, would increase followers’ trust under conditions of different followers’ interpretations about leader humility which represents followers’ rational choice during trust development. Summarizing the above statements, we propose the theoretical model illustrated in Figure 1.

## Theory and Hypotheses

### Humble Leader Behaviors: Humility in Leadership Studies

The general construct of humility has a rich background in theology and philosophy, and it has been categorized as a temperance virtue that guards against excess (Peterson and Seligman, 2004). Although the virtue of humility has rich historical roots, conceptualizations of humility vary significantly across philosophical, theological, and psychological perspectives. According to the literature, there is no clear consensus about precisely what kind of construct humility is. In addition to defining humility as a “virtue” (Peterson and Seligman, 2004), this concept has variously been described as a “relationship-specific personality judgment” (Davis et al., 2011); a “personality trait” (Ashton and Lee, 2009); a “state” (i.e., characterized by reduced self-focus; Kruse et al., 2014); an “emotion” (Saroglou et al., 2008); and an “accurate assessment of one’s abilities and strengths” (Tangney, 2000). Facing such various ways to operationalize the humility construct, scholars summarized that humility is a complicated concept that consists of multiple psychological structures: thoughts, feelings, and behaviors (Weidman et al., 2018).

Recognizing the complexity of the humility concept, we realize that when introducing humility to leadership studies, it is necessary to specifically clarify what psychological structures of humility we are interested in. In response to the international recommendation that management study should return to management practice, Owens and Hekman (2012) proposed the humble leadership theoretical model and called for studies to focus on humble leader behavior. According to Owens and Hekman (2012), aside from valuing leaders that possess the humility trait, we should understand how humble leadership looks like in terms of an overall leadership posture and what behaviors it involves. Based on the humble leadership framework (Owens and Hekman, 2012), Owens et al. (2013) focused their interests in the behavioral aspects of humility and defined expressed humility as an interpersonal characteristic that emerges in social contexts that connotes the following behaviors: (a) a manifested willingness to view oneself accurately, (b) a displayed appreciation of others’ strengths and contributions, and (c) teachability. Expressed humility comprises a pattern of humble behaviors that occur in interpersonal interactions and is therefore observable by others.

Through expressions of humility, leaders convey a tendency to approach interpersonal interaction with their followers, driven by strong learning motives, which in turn positively influences employees (Owens and Hekman, 2012). However, leader humility is conceptually distinct from other established leadership styles. For example, both authentic and humble leaders have accurate self-views, but these two kinds of self-views serve different motives. Authentic leaders’ accurate self-views serve for their striving for consistency between personal values and behaviors (Avolio and Gardner, 2005), while humble leaders’ accurate self-views mainly regarding self-weaknesses serve for their innate drive to learn (Owens and Hekman, 2012). Besides, although participative and humble leaders both exhibit openness to others’ ideas, participative leaders emphasize sharing power during decision making (Huang et al., 2010), while humble leaders value others’ opinions because they can appreciate and learn from other people’s strengths (Owens and Hekman, 2012). Furthermore, though servant leaders and humble leaders equally value the development of followers, they utilize different ways to achieve their goals. Servant leaders develop followers by serving them (Barbuto and Wheeler, 2006) while humble leaders stimulate followers’ development by modeling self-growth (Owens and Hekman, 2012).

### Followers’ Capability, Leader Expressed Humility, and Follower Trust

Rational choice is the fundamental consideration for human beings to behave properly during interactions with others (Coleman and Fararo, 1992). Rational choice theory posits that people engage in conscious cost-benefit calculations such that they maximize the benefits and minimize the costs of their actions (Hechter and Kanazawa, 1997). Several applications of rational choice theory in the field of organizational research have suggested that a strong motivation toward chasing maximized utility and the aversion of potential risk play similar important roles while influencing ones’ organizational behavior decisions (Morrell, 2004; Werbel and Balkin, 2010).

Many studies examining organizational humble behaviors have pointed out that people’s sensitivity to potential behavioral risk (e.g., exposing one’s weakness in unsafe circumstances while engaging in humble behaviors) will inhibit expressions of humility (Owens, 2009). However, in such discussions, the role played by benefit calculations of humble behaviors has been neglected. Indeed, individuals can confer adaptive advantages in terms of personal growth and social support by expressing humility (Tong et al., 2016). By behaving humbly, leaders show their appreciation of others’ strength and expect to benefit and learn from others (Owens and Hekman, 2012). Thus, when they have high expectation for benefits brought by humble behaviors, leaders will mostly be motivated to express humility as a rational choice.

Leaders’ expectations regarding benefits from interpersonal humble behaviors could be largely determined by features of their humility expression targets. More specifically, in terms of reasoning, leaders’ humble behavior can be stimulated by highly beneficial targets. Thus, as the main interaction targets of leaders in the workplace, followers can serve as predictors of humble leader behavior. Previous studies found that when followers have high capability, they will be perceived as persons of high utility by the leaders. Thereupon, leaders would initiate positive interaction with such followers (Tepper et al., 2011; Walter et al., 2015). Hence, we could assume that leaders would expect greater returns by interacting with followers who possess high capabilities, so, in turn, leaders get more motivated to behave in a humble manner. We therefore propose that follower capability would positively impact leaders to develop and express humility by awarding intrapersonal and interpersonal benefits to themselves (Tong et al., 2016).

Turning to intrapersonal aspects, expressed humility can help leaders to foster learning and personal growth (Nielsen and Marrone, 2018). The enactment of humble behaviors indicates the potential self-transcending process of leaders (Morris et al., 2005). Transcending oneself in the concept of humility indicates that individuals are aware of something greater than the self, they are less concerned with themselves and realize that they only play small roles in a vast universe (Morris et al., 2005; Ou et al., 2014b). According to Owens et al. (2013), humility expression allows one to transcend the comparative–competitive response when interacting with others and instead acknowledge and admire the strengths and contributions of others without feeling threatened by them (Exline and Geyer, 2004). The humble behavior enacted by leaders reveals that leaders are able to identify in others valuable resources for learning and personal growth. Therefore, the innate motivation behind gaining the intrapersonal benefits of humility (learning and personal growth) is one’s eagerness to learn (Owens et al., 2011). While ones’ motivation to learn is decided mostly by the learning object’s ability, i.e., people become more motivated to learn from others who are experts or are highly competent and able (Augustinova et al., 2005). Following the theory of rational choice, when the gain expectation is high, individuals exhibit a greater propensity for action (Hechter and Kanazawa, 1997). When the followers are perceived to be capable and talented, the leaders will expect to learn more from them, which in turn will trigger the need for the leader to express humility.

Besides, from an interpersonal perspective, humble individuals gain a lot from reciprocal interpersonal relationships by actively facilitating altruistic behaviors and inhibiting the desire to show superiority during interpersonal interactions (Davis et al., 2011; Van Tongeren et al., 2014). When the followers are perceived to be capable, the leader would be more likely to initiate positive behavior to build high-quality relationships with them (Whitener et al., 1998). It is very important to gain insights from capable followers, because: (a) followers with the right skills and foresight can be good aides to leaders, and (b) leaders expressing humility can promote better talent management. Here, talent is viewed as the ability of an employee to become a star performer (Lewis and Heckman, 2006). Those leaders exhibiting humility can satisfy employees’ need for membership identification (i.e., make them feel valued). This facilitates retention of capable employees (Groysberg et al., 2011). As such, this helps develop high loyalty to the organization among the followers (Call et al., 2015). Based on the above statements, we can assume that leaders get a lot in return by acting humbly, when surrounded by capable followers. Therefore, we propose:

***Hypothesis 1: Leader-perceived capabilities of followers are positively related to leader humility.***

By pursuing the intrapersonal and interpersonal benefits of humility expressing behaviors, leaders should be able to improve their management ability (Vera and Rodriguez-Lopez, 2004) and foster good relationships with their followers (Exline and Geyer, 2004). When leaders exhibit favorable personal characteristics and interpersonal relationships, followers can easily develop trust in their leaders (Mayer et al., 1995). Moreover, when leaders demonstrate awareness to their own limitations, followers would be able to shape authentic views of their leaders. This would encourage followers to develop trusted relationships with leaders (Owens et al., 2011). Therefore, we propose that:

***Hypothesis 2: Leader humility is positively related to follower trust.***
  ***Hypothesis 3: Leader-perceived capabilities of followers increase follower trust indirectly through the effect of leader humility.***

### Rational Attribution in Trust Development

As mentioned above, by reaping the intrapersonal and interpersonal benefits brought by humility expression, leaders can facilitate the generation of follower trust (Mayer et al., 1995; Owens et al., 2013). However, from the viewpoint of rational choice with respect to trust building, the trustor’s rational evaluation toward the trustee plays a central role in fostering trusting relationships (Hardin, 1993; Weber et al., 2004; Lewicki et al., 2006). Similarly, Mayer et al. (1995) defined trust as one’s willingness to be vulnerable to the actions of another party, indicating trusting behavior, *per se*, could be risky since the consequences of trust violation could be quite traumatic, e.g., psychological contract breach (Elangovan and Shapiro, 1998). This suggests that trust should be viewed as a rational-choice behavior (Hardin, 1993; Lewicki et al., 2006). In short, to avoid the cost of trusting mistakenly, trustors should evaluate and make inference about trustees’ behavioral intentions rationally (Weber et al., 2004; Whipple et al., 2013). When individuals make positive attributions about trustees’ intentions, they would be more likely to generate trust toward others. Otherwise, it is not only possible that trust would not be generated, but the one developed could also be eroded (Elangovan et al., 2007).

Since expressed humility is malleable and changeable in light of different situations, followers may have different interpretations about leader expressed humility (Owens et al., 2013), which will influence building trust toward the leader. Halbesleben et al. (2010) pointed out that individuals could interpret and ascribe different motives to others’ behaviors, such as sincere motives and instrumental motives. Although humility has been regarded as a virtue for a long time (Tangney, 2000; Exline and Geyer, 2004; Owens and Hekman, 2012), scholars have also examined instrumental motives behind expressing humility—since humility is highly related to impression management and social desirability (Rowatt et al., 2006; Davis et al., 2011). Thus, when others observe this kind of behavior, humility expression can be said to be containing certain instrumental consideration. When humble behavior on the part of the leader is seen as an act that benefits other members of the team, followers will exhibit a greater propensity for trust, and when humility expression is considered to be serving the leader’s instrumental consideration, such as impression management, followers will be suspicious of the true motives behind leaders’ behaviors (Bass and Steidlmeier, 1999; Li et al., 2017) and any existing trust will be eroded (Elangovan et al., 2007).

Drawing on the theory of rational choice of trust building (Hardin, 1993; Lewicki et al., 2006), we propose that followers’ interpretations about leader humility will influence the relationship between leader humility and follower trust. Similarly, Dasborough and Ashkanasy (2002) found that when followers attribute transformational leadership behaviors to impression management, the transformational leadership will be perceived to be fake rather than sincere (Dasborough and Ashkanasy, 2002). Besides, Owens and Hekman (2012) proposed that leader humility can only provoke positive outcomes when followers perceive leaders’ humility as “sincere humility” rather than “instrumental humility” (Owens and Hekman, 2012). Therefore, we propose that:

***Hypothesis 4a: When followers attribute leader humility as serving performance enhancement motives, there would be a stronger positive relationship between leader humility and follower trust.***
  ***Hypothesis 4b: When followers attribute leader humility as serving impression management motives, the relationship between leader humility and follower trust would only be mildly positive.***

### Leader and Employee Rationality

Active leadership styles indicate that effective leadership is not only a personality type but also a function of a leader’s ability to read contextual cues and make rational behavioral decisions (Ewen et al., 2013). While seeking to promote better interaction with followers, leaders should adjust their behaviors in accordance with the characteristic features of the followers. Drawing on rational choice theory (Coleman and Fararo, 1992; Lewicki et al., 2006), the foregoing considerations, taken together, point toward the totality of interactional processes prevailing between capable followers and humble leaders; they also highlight the interpersonal characteristics of leader humility (see Figure 1). On the one hand, as a rational choice for the leader, the more capable the followers are perceived by their leader, the more likely the leader will be able to express humility (Hypothesis 1). On the other hand, again as a rational choice for the followers and depending on their attributed motives of leader humility, they will conditionally develop trust in their ‘humble’ leaders (Hypothesis 4a and 4b). Therefore, taking the rational choices of the leader and the followers together, we propose a pattern of conditional effects of followers’ inferring leader humility motives as indirect effects between leaders’ perceived capabilities of followers and follower trust via leader humility. Specifically, we propose that:

***Hypothesis 5a: Followers inferring leader humility motives for performance enhancement moderates the indirect effect of leader-perceived capability of followers on follower trust as mediated by leader humility. The indirect effect is stronger when followers infer leader humility as serving performance enhancement motives.***
  ***Hypothesis 5b: Followers inferring leader humility motives for serving impression management moderates the indirect effect of leader-perceived capability of followers on follower trust as mediated by leader humility. The indirect effect is weaker when followers infer leader humility as serving impression management motives.***

## Materials and Methods

### Participants and Procedure

This research was conducted in accordance with the recommendations of the Science & Technology Research Office of HuaZhong University of Science and Technology. Since the study did not involve human clinical trials or animal experiments and there were no unethical behaviors in the research process, ethics approval was not required as per institutional and national guidelines and regulations. The informed consent of the participants was implied through survey completion. In China, the government has a very good relationship with companies. Thus, one of the authors first contacted a Mayor and then distributed the questionnaires directly at the workplace with the support of the Mayor. The leaders and the followers were matched in our research design to reduce common method bias (Podsakoff et al., 2003). In the first page of our questionnaire, consent was presented to inform participants that they were completely free to join or drop out of the survey. Only those who were willing to participate were recruited. Each respondent was provided with a letter to introduce the purpose, a paper questionnaire and a gift worth about 2 dollars. Data was collected from 13 companies located in Mainland China. Industries varied from manufacturing industry (6 companies), real estate (3 companies) to high-tech industry (4 companies) among these 13 companies.

A time-lagged research design was adopted to test our theoretical model. At Time 1, we measured perceived capability of followers from the leader. Meanwhile, followers were asked to report leader humility and inferred motives of leader humility. At Time 2 (about 7 weeks later), we measured follower trust toward their leader by the followers. Questionnaires were directly distributed and collected at the workplace. At Time 1, we distributed the questionnaires to 72 leaders and 350 followers, collected data from 350 leader-follower dyads. After Time 2, we received well completed questionnaires from 64 leaders and 278 followers and collected data from 278 leader-follower dyads, with a response rate of 89 and 82% for leaders and followers respectively. Among leaders, 48% of them were men, with an average age of 39.0 years (SD = 8.47). Among followers, 56% of them were men, with an average age of 31.6 (SD = 8.38).

### Measures

All English-based measures were translated into Chinese according to the “translation/back-translation” procedures. A Likert-type scale ranging from one (*strongly disagree*) to seven (*strongly agree*) was used for all the measures.

#### Leader Humility (Follower-Rated at Time 1)

We used a nine-item scale developed by Owens et al. (2013) to measure leader humility (α = 0.91). One sample item was: “This person actively seeks feedback, even if it is critical.”

We constructed leader humility as an individual-level variable in our research to achieve the match between theory and model testing (Van Maanen et al., 2007; Wang and Cheng, 2010). Theoretically, the rational choice between leader and followers happens over the course of interaction within the leader-follower dyad; thus, it is appropriate to conduct analysis at an individual level. Similarly, previous research conducted on leader-follower dyad interaction also adopted single level analysis (i.e., Vecchio et al., 2010; Peus et al., 2012).

#### Inferred Motives of Leader Humility (Follower-Rated at Time 1)

We adopted Allen and Rush’s (1998) fourteen-item scale to measure inferred motives of leader humility. Lam et al. (2007) used this scale to measure leader-inferred followers’ motives of impression management and performance enhancement. Since there were no established measures for inferred motives of leader humility, we modified this scale to measure follower-inferred leaders’ motives for expressing humility by reversing the evaluation object (i.e., replace “the follower…” with “the leader…” in each item). Seven items were used to measure inferred motives of impression management (α = 0.91). The remaining seven items were used to measure inferred motives of performance enhancement (α = 0.91). We measured inferred motives of leader humility directly after the measurement of leader humility in the questionnaire. Followers were asked to evaluate “What do you think your leader’s motive is for displaying the behaviors from the previous question?” One sample item of inferred motives for performance enhancement was “Desire to best fulfill his or her responsibilities.” One sample item for inferred motives for impression management was “Desire to create a good impression.”

#### Follower Trust (Follower-Rated at Time 2)

We used Jarvenpaa and Leidner (1999)’s four-item scale to measure followers’ trust in leader (α = 0.81). Followers were asked to evaluate their trust relationship toward their leader. Example items included “I could rely on my leader” and “Overall, my leader is very trustworthy.”

#### Leader-Perceived Capability of Follower (Leader-Rated at Time 1)

We used two standard measurements to measure leaders’ perceptions of followers’ capability relatively (i.e., perceived follower advantage) and absolutely (i.e., perceived star employee). For *relative standard:* perceived follower advantage was measured with five items by asking leaders to compare themselves with their followers in the domains of professional expertise, including knowledge, skills, social recognition, meta-cognitive knowledge and flexibility (Johanna and Van der Heijden, 2000). One sample question was: “How do you compare with this follower in the knowledge dimension?” (α = 0.91). For *absolute standard:* perceived star employee was measured by asking leaders how they view their followers as outstanding. The star employee was measured using the five-item scale developed by Long et al. (2015). Leaders were asked to rate every subordinate to so their star employee could be identified. One sample question was: “This employee has what it takes to go far in my organization.” (α = 0.91).

#### Control Variables

To exclude the potential confounding effect of follower gender, age, tenure and followers’ perception of leader-follower interaction frequency on relationship between leader’s perception of follower capability and leader humility (Ou et al., 2014a), we controlled follower gender, age, organization tenure, and follower-perceived leader-follower interaction frequency. For follower gender, we used a dummy variable with 0 representing female and 1 representing male. Moreover, the outcome of humility expression (e.g., follower trust) and leaders’ behavioral intentions toward humility expression will be affected by leaders’ humility trait. Therefore, we also treated the leader humility trait as another control variable. We used the scale developed by Ashton and Lee (2009) to measure leaders’ humility trait. The scale contains five items, example include “I wouldn’t use flattery to get a raise or promotion at work, even if I thought it would succeed” (α = 0.88).

## Results

### Descriptive Results

The means, standard deviations, and correlations for all variables are shown in Table 1. Leader-perceived follower advantage and star employee were both significantly related to leader humility (*r* = 0.13, *p* < 0.05; *r* = 0.12, *p* < 0.05, respectively). Follower trust was significantly related to leader humility (*r* = 0.30, *p* < 0.01).

**TABLE 1:** Descriptive results.

**Variable**	**Mean**	**SD**	**1**	**2**	**3**	**4**	**5**	**6**	**7**	**8**	**9**	**10**
1. Follower gender	0.57	0.50										
2. Follower age	31.69	8.42	–0.27^∗∗^									
3. Follower tenure	5.38	6.97	–0.12	0.64^∗∗^								
4. Interaction Frequency	1.41	0.70	0.05	0.10	0.01							
5. Leader humility trait	5.23	0.93	0.16^∗∗^	–0.02	–0.05	0.05						
6. Star Employee	4.95	1.12	–0.03	–0.06	0.02	–0.01	0.07					
7. Perceived followers’ advantage	3.43	1.14	−0.13^∗^	0.08	0.01	–0.09	0.18^∗∗^	0.16^∗∗^				
8. Leader humility	6.03	0.90	–0.03	0.14^∗^	0.15^∗^	0.01	0.16^∗∗^	0.12^∗^	0.13^∗^			
9. IMPE	5.85	0.90	–0.01	0.06	0.13^∗^	–0.33^∗∗^	0	0.08	0.03	0.09		
10. IMIM	4.44	1.30	0	–0.11	–0.04	–0.10	0.01	–0.01	0.20^∗∗^	–0.18^∗∗^	0.10	
11. Follower trust	5.59	0.99	–0.05	0.22^∗∗^	0.08	0	0.21^∗∗^	0.12^∗^	0.08	0.30^∗∗^	0.04	–0.22^∗∗^

*N dyads = 278. ^∗^*p* < 0.05, ^∗∗^*p* < 0.01. IMIM refers to inferred motives for impression management. IMPE refers to inferred motives for performance enhancement.*

### Confirmatory Factor Analyses

We first adopted the confirmatory factor analysis using Amos 22.0 to verify convergent and discriminant validity of the constructs in our model. The measurement model was composed of six latent factors with 38 indicators (six items for perceived follower advantage, five items for star employee, nine items for leader humility, four items for trust, seven items for inferred motives for impression management, and seven items for inferred motives for performance enhancement). The standardized residual matrix and modification indices showed that the initial six-factor measurement model was required for improvement in model fit.

Given the relatively small sample size, composite formation techniques were used to create item parcels for measuring constructs in our study. The most important principle for item parceling is that the use of parceling depends on the unidimensionality of the items being combined (Bandalos and Finney, 2001). Following such a principal, Landis et al. (2000) posited that one rational judgment about which items should be assigned to the same parcel is based on existing theory or previous definition about the concept. Leader humility expression was measured by nine items representing three dimensions: willingness to see the self accurately, appreciation of others’ strengths, and teachability (Owens et al., 2013). Thus, a content-oriented strategy was used for leader humility expression to provide an adequate representation of the three dimensions. Each subset of items for the dimensions was averaged into a new single item, resulting in three parcels. In addition, Landis et al. (2000) also suggested that when the construct is a unidimensional scale, researchers can apply a data-driven method such as exploratory factor analysis to inspect whether the remaining items can be classified into different parcels. Therefore, we ran exploratory factor analysis (EFA) for each construct to see if any constructs could be divided into different dimensions for further item parceling (Bandalos and Finney, 2001).

After item parceling, we conducted the procedure by inspecting the modification indices of EFA results. Final results in Table 2 showed that our initial six-factor model had a superior model fit over other alternative models (χ^2^ = 750.01, *df* = 305, CFI = 0.93, TLI = 0.91, RMSEA = 0.07, Δχ^2^ = 633.78), confirming the discriminant validity of these constructs in our model.

**TABLE 2:** Confirmatory factor analyses results.

**Model**	**χ^2^**	**D*f***	**Δχ^2^**	**RMSEA**	**TLI**	**CFI**	**NFI**
Six-factor model (HL; PA; STE; IMIM; IMPE; T)	750.01	305	—	0.07	0.91	0.93	0.89
Five-factor model (HL; PA + STE; IMIM; IMPE; T)	1383.79	310	633.78^∗∗^	0.11	0.79	0.83	0.79
Four-factor model (HL; PA + STE; IMIM + IMPE; T)	1653.96	314	270.17^∗∗^	0.12	0.74	0.78	0.75
Three-factor model (HL + T; PA + STE; IMIM + IMPE)	1959.08	317	305.12^∗∗^	0.14	0.68	0.74	0.70
Two-factor model (HL + T + PA + STE; IMIM + IMPE)	2541.04	319	581.96^∗∗^	0.16	0.57	0.64	0.61
One-factor model (HL + PA + STE + IMIM + IMPE + T)	3033.204	320	492.16^∗∗^	0.18	0.48	0.56	0.54

*HL refers to leader humility. PA refers to perceived followers’ advantage. STE refers to star employee. IMIM refers to inferred motives for impression management. IMPE refers to inferred motives for performance enhancement. T refers to trust. “ + ” refers to combine. ^∗∗^*p* < 0.01.*

### Hypothesis Testing

Hypothesis 1 posited that both leader-perceived follower advantage and star employee were positively related to leader humility. We separately regressed leader-perceived follower advantage and star employee on leader humility. The results from Model 1b and c (see Table 3) showed that leader-perceived follower advantage was positively related to leader humility (β = 0.15, *p* < 0.05), and star employee was also positively related to leader humility (β = 0.12, *p* < 0.05). Hence, hypothesis 1 was supported.

**TABLE 3:** Regression analyses results.

**Variable**	**Leader humility**			**Follower trust**			
		
	**Model 1a**	**Model 1b**	**Model 1c**	**Model 2a**	**Model 2b**	**Model 2c**	**Model 2d**
**Intercept**	4.44^∗∗∗^	3.87^∗∗∗^	4.12^∗∗∗^	4.29^∗∗∗^	4.17^∗∗∗^	4.27^∗∗∗^	4.12^∗∗∗^
**Control variables**							
Follower Gender	–0.03	–0.02	–0.01	–0.03	–0.03	–0.04	–0.03
Follower Age	0.13	0.15^+^	0.13	0.17^∗^	0.17^∗^	0.17^∗^	0.18^∗^
Follower Tenure	0.06	0.04	0.06	–0.04	–0.04	–0.05	–0.05
Follower perceived leader-follower interaction frequency	0.07	0.07	0.10	–0.06	–0.05	–0.06	–0.04
Leader humility trait	0.19^∗^	0.18^∗^	0.15^∗^	0.16^∗^	0.17^∗^	0.16^∗^	0.17^∗^
**Independent Variables**							
Perceived followers’ advantage			0.15^∗^				
Star Employee		0.12^∗^					
Leader humility				0.28^∗∗∗^	0.22^∗∗∗^	0.27^∗∗∗^	0.22^∗∗∗^
IMIM					−0.15^∗^		−0.16^∗^
IMPE						0.00	0.03
Leader humility^∗^IMIM					−0.14^∗^		−0.14^+^
Leader humility^∗^IMPE						0.09	0.06
Leader humility^∗^IMIM^∗^ IMPE							0.04
**R^2^**	0.07^∗∗^	0.09^∗∗^	0.09^∗∗^	0.16^∗∗^	0.20^∗∗^	0.16^∗∗^	0.21^∗∗^
**ΔR^2^**	0.07^∗∗^	0.02^∗^	0.02^∗^	0.16^∗∗^	0.04^∗∗^	0.00	0.05^∗^
**ΔF**	3.50^∗∗^	3.57^∗^	4.86^∗^	6.76^∗∗^	5.79^∗∗^	0.95	2.56^∗^

*N dyads = 278. Standardized regression coefficients reported. +*p* < 0.1; ^∗^*p* < 0.05; ^∗∗^*p* < 0.01; ^∗∗∗^*p* < 0.001. IMIM refers to inferred motives for impression management. IMPE refers to inferred motives for performance enhancement.*

**FIGURE 2:**
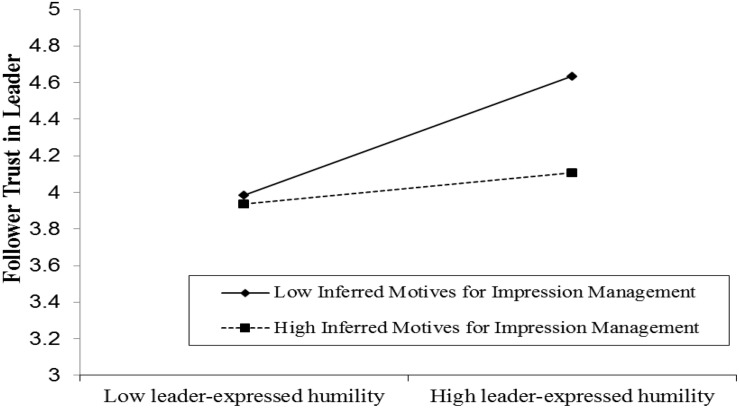
Moderating effect of inferred motives for impression management on the relationship between leader-expressed humility and follower trust.

Results of Model 2a in Table 3 showed that leader humility was positively related to follower trust (β = 0.28, *p* < 0.001), supporting Hypothesis 2. Hypothesis 3 proposed that leader-perceived follower advantage and star employee would indirectly increase follower trust by invoking leader humility. To test this indirect effect, we used bias-corrected bootstrapping techniques (1000 replications). The results showed that leader-perceived follower advantage had an indirect effect on follower trust via leader humility (indirect effect = 0.04, 95% CI = [0.01, 0.09], excluding zero), and the mediating effect of leader humility could also be found in the relationship between star employee and follower trust (indirect effect = 0.03, 95% CI = [0.01, 0.07], excluding zero). Hence, Hypothesis 3 was supported. Results from Model 2b and Model 2c in Table 3 showed that the moderating effect of inferred motives for impression management was significant (β = −0.14, *p* < 0.05), while the moderating effect of inferred motives for performance enhancement was not significant (β = 0.09, n.s.). As Figure 2 presented, when followers highly attribute leader humility as serving impression management motives, the positive relationship between leader humility and follower trust vanished. Hence, hypothesis 4b was supported and hypothesis 4a was not supported.

Other than this, we also used bootstrapping techniques to test the moderated mediation effect. Table 4 showed the indirect effect under high and low levels of moderation. The results showed that inferred motives for impression management moderated the mediating effect of leader humility between perceived follower advantage and follower trust (index of moderated mediation = −0.02, 95% CI = [−0.06, −0.00], excluding zero), and this result remained significant when we changed the independent variable into star employees (index of moderated mediation = −0.02, 95% CI = [−0.05, −0.00], excluding zero). Meanwhile, moderating mediation was not significant when we changed the moderator into inferred motives for performance enhancement motives (independent variable for perceived follower advantage: index of moderated mediation = 0.01, 95% CI = [−0.01,0.05], including zero; independent variable for star employee: index of moderated mediation = 0.01, 95% CI = [−0.01, 0.05], including zero). Hence, hypothesis 5b was supported, while hypothesis 5a was not supported.

**TABLE 4:** Moderated mediating effects.

**Moderator**	**Leader-perceived follower advantage (X) → Leader humility(M) → Follower trust(Y)**			**Star employee (X) → Leader humility(M) → Follower trust(Y)**		
		
	**Indirect effect**	**SE**	**95% CI**	**Indirect effect**	**SE**	**95% CI**
**Inferred motives for impression management**						
Low (−s.d)	0.05	0.03	[0.01, 0.13]	0.04	0.03	[0.01, 0.10]
Mean	0.03	0.02	[0.01, 0.08]	0.02	0.02	[0.01, 0.06]
High (+ s.d)	0.01	0.02	[−0.02, 0.06]	0	0.01	[−0.01, 0.04]
**Inferred motives for performance enhancement**						
Low (−s.d)	0.03	0.02	[0, 0.09]	0.02	0.02	[−0.01, 0.08]
Mean	0.04	0.02	[0.01, 0.09]	0.03	0.02	[0, 0.08]
High (+ s.d)	0.05	0.03	[0.01, 0.12]	0.04	0.03	[0, 0.11]

*CI refers to confidence interval.*

## Discussion

The present study has investigated why leaders often express humility and how this matters to followers based on rational choice theory. We have found that when the leader perceives that his/her followers possess capabilities of a high order, the leader would be more likely to express humility. We have also found that leader humility could promote trusting relationships among the followers toward the leader. Finally, we have presented the total process underpinning dyadic level leader-follower interactions. By making their abilities more visible to their leader, followers can enhance leader-expressed humility, and, in turn, through leaders’ humility expressions, followers can develop greater trust in their leaders. This interaction hinges on followers’ positive inferences about the motives behind the leader’s expressions of humility, that is, when followers interpret leader humility as serving impression management motives, it is less likely that such leader behavior will increase follower trust.

However, as for inferring leader humility, with regard to performance enhancement motives in the relationship between leader humility and follower trust, we did not find a moderation role of inferred leader humility motives. Initially, we thought that this result was beyond expectation, but reasonable. Drawing from rational choice theory (Lewicki et al., 2006), we found that the attribution of behavior motives is more to do with identifying a mismatch between behavior and intention. Such a matching process will help individuals avoid trusting the wrong person (Elangovan et al., 2007). However, the actual source of increase or decrease of trust is usually more related to the characteristics of the trustees (Mayer et al., 1995). Thus, compared to leaders’ humility characteristics, followers’ attribution of leader humility motives may have less impact on followers’ trust building toward the leader. Additionally, individuals are more sensitive about negative information and events (“negative bias”, Rozin and Royzman, 2001), which may serve as an explanation for the untested hypothesis. We strongly suggest future research to dig further into this issue.

### Theoretical Implications

The present research has contributed to leadership and leader humility literature in several ways. Firstly, our study is the first to examine situational predictors of leader humility. By treating humility expression targets as possible antecedents of leader humility, the present study has provided a novel understanding of why leaders express humility. Most previous studies on leader humility have focused on its positive outcomes (Owens et al., 2013; Ou et al., 2014b, 2017; Owens and Hekman, 2016); few examined the antecedents of leader humility. Moreover, the few research scholars who had evaluated individual differences such as personal traits or life experience as the antecedents of humility, have treated humble leadership as a trait-relevant leadership style (Morris et al., 2005; Owens, 2009). Although many scholars have proposed that contextual factors such as safe climate could trigger greater leader-expressed humility (Owens, 2009), empirical research examining the situational predictors of leader humility is scarce. Drawing from rational choice theory, this research has found that follower capability would trigger leaders to express humility. Thus, this research has been able to explain leaders’ expressions of humility.

Secondly, our study has contributed to leadership literature by emphasizing the follower-centric view which values followers as a critical factor that could shape leaders’ behavior and influence effectiveness of leadership. Our review of the leadership literature has noted that most previous leadership studies had endorsed the leader-centric view (i.e., followers are only considered as recipients or moderators of leadership) while ignoring the follower-centric view (i.e., followers can be seen as “constructors” of leadership) (Howell and Shamir, 2005; Uhl-Bien et al., 2014). The present study is the first to empirically test the followers’ role in affecting the processes underlying humble leadership. Firstly, followers play an important role in shaping expressions of humility on the part of the leader; specifically, followers could have the power to trigger more leader humility when their abilities become salient to their leaders. Secondly, followers’ interpretations play a key role in affecting the outcomes of humble leadership. Like many other studies related to positive leadership, e.g., transformational leadership (Dasborough and Ashkanasy, 2002), positive outcomes could not be guaranteed if followers interpret leaders’ behavior as being distorted in some way. Similarly, we found that leader humility cannot lead to greater follower trust if it gets interpreted as serving impression management motives.

Thirdly, the present study has furthered the understanding of leader humility by integrating rational choice theory and leadership theory. Just as the Confucian proverb says “haughtiness invites loss while humility brings benefits,” humility has been credited with bringing intrapersonal and interpersonal benefits (Cai et al., 2010). Consistent with rational choice theory, the present research has found that leaders’ perception of followers’ capabilities positively influences the leader’s humility expressing behavior since the leader can benefit more by being humble with capable followers. This is in consonance with the opinion that leaders would exhibit more positive behaviors to outstandingly good followers (Uhl-Bien et al., 2014). Furthermore, we have proposed and found that followers’ rational attribution about leader humility would influence the relationship between leader humility and follower trust. Therefore, by integrating rational choice theory with the construct of leader humility, we have been able to obtain a deeper understanding of the interaction between humble leaders and their followers.

### Practical Implications

For managerial practice, we hope both leaders and followers would pick up some insights during the daily workplace interactions. For followers, they should realize the malleability of leader-expressed humility. This might provide two pieces of advice for promoting better interactions between followers and leaders. Firstly, followers should realize that they have a role in stimulating positive behavior on the part of the leader. By actively performing better at their respective jobs, followers could be appreciated by others at the workplace (including leaders). They should also realize that leaders exhibit certain behaviors based on some instrumental calculation, suggesting that followers should seek to inform themselves more assiduously before arriving at a final evaluation of their leader (Tepper et al., 2011).

As for leaders, although they may expect positive outcomes when they constantly express humility toward their followers, they should reflect upon the sincerity of their own humility expressions, in case the outcomes fail to meet expectations. Leaders should be aware of the importance of being more in service of the followers and the group rather than about themselves. However, if leaders constantly put up an act but in reality look after their own interests, their true intentions behind their behaviors would soon become apparent to their followers and, in time, erode existing trust. By sending feelers that their intentions and behavior have been consistent (Simons, 2002), leaders can protect themselves from being perceived as hypocritical.

What is also worth noting is that one limitation of this study is that we reported small effect sizes of follower competence on leader humility expression, which raises the question of whether these effects have meaningful implications for practice in management. The answer to this question is “yes.” Firstly, our small effect sizes are comparable to some previous studies of leader humility (Qian et al., 2018). Secondly, despite the small sizes, we obtain such effects after ruling out the influence of leaders’ humility trait. These results are practically meaningful because it indicates that we can cultivate humble leadership (e.g., humility-expressing behavior) through shaping the situational factors. Different from previous studies that only valued individual differences as antecedents of leader humility, our study indicates that organizations can create a better environment to trigger positive humble leader behaviors rather than cultivating humble leadership largely depending on the selection of leaders (e.g., selecting leaders with a high level of humility).

### Limitations and Future Research

Firstly, although we utilized matching data analysis and multi-wave data collection as a method to verify our hypotheses, the sampling data could not offer causal inferences about our hypotheses. We recommend a longitudinal study to evaluate the actual causal relationship. Secondly, our study was conducted in China, where acting humbly is among the cultural norms and so individuals are suggested not to show off (Hwang, 1982; Kurman and Sriram, 1997). It is possible that people in such situations might be acting humbly against one’s true will and feelings. Further, the Chinese might be having varying interpretations of others’ humility. It is therefore not clear to what extent our results can be generalized or if our findings can be applicable to the Western context. We advocate further research to explore whether and how cultural differences influence the model proposed in this research—for example, whether a leader with higher dependent self-construal who values more harmonious interpersonal relationship will express more humble behaviors. Thirdly, the present study has left a hypothesis implicit in multiple empirical studies, namely, followers with higher capability would be perceived as having high utility by leaders. However, as many researchers have argued, there could be the possibility that when followers have high capability, leaders would sense both utility and be personally threatened at the same time (Khan et al., 2018). Finally, we acknowledge that rational consideration is one possible angle to understand the situational predictors of leaders’ humility expression. Beyond that, we think leaders’ less rational emotional perception can also be situational predictors of leader humility. These limitations also point to possible future directions for humble leadership studies.

## Conclusion

The interpersonal definition of leader humility implies that humility expressions represent one kind of interpersonal interaction between humility actors and humility recipients. In consonance with the widely held opinion that leadership studies should simultaneously value the roles of leader and his/her followers, our study has combined the consideration of the actor and the recipients. We presented the rational exchange process in a dyadic leader-follower relationship and found that a leader’s rational choice to develop and express humility depends on followers’ capability. Conversely, a followers’ rational choice to trust a humble leader depends on the attribution of the motives for the leader’s humility. We hope our work has led to a deeper understanding of the utility of leader humility by highlighting the interpersonal perspective.

## Data Availability

The raw data supporting the conclusions of this manuscript will be available to any qualified researcher from the corresponding author upon request.

## Author Contributions

XC wrote the manuscript and analyzed the data. JY and WZ  wrote the manuscript.

## Conflict of Interest Statement

The authors declare that the research was conducted in the absence of any commercial or financial relationships that could be construed as a potential conflict of interest.
